# Isolation of saprophytic filamentous fungi from avian fecal samples and assessment of its predatory activity on coccidian oocysts

**DOI:** 10.1038/s41598-023-36120-5

**Published:** 2023-06-02

**Authors:** João Lozano, Mariana Louro, Cristina Almeida, Ana Cláudia Victório, Pedro Melo, João Paulo Rodrigues, Manuela Oliveira, Adolfo Paz-Silva, Luís Madeira de Carvalho

**Affiliations:** 1 CIISA—Centre for Interdisciplinary Research in Animal Health, Faculty of Veterinary Medicine, University of Lisbon, Avenida da Universidade Técnica, 1300-477 Lisbon, Portugal; 2 Associate Laboratory for Animal and Veterinary Sciences (AL4AnimalS), 1300-477 Lisbon, Portugal; 3 Exoclinic – Clínica Veterinária de Aves e Exóticos, Quinta de Santo António, 1495-049 Miraflores, Portugal; 4 Vetnatura – Serviços Veterinários, Lda., Calçada de Palma de Baixo, 1600-176 Lisbon, Portugal; 5 Bark – Biopark Barquinha, 2260-999 Vila Nova da Barquinha, Portugal; 6 Control of Parasites Research Group (COPAR, GI-2120), Department of Animal Pathology, Faculty of Veterinary, University of Santiago de Compostela, 27142 Lugo, Spain

**Keywords:** Microbiology, Fungi, Parasitology

## Abstract

Fungal strains used in the biocontrol of animal gastrointestinal parasites have been mainly isolated from pasture soil, decaying organic matter, and feces from herbivores and carnivores. However, their isolation from birds and assessment of predatory activity against avian GI parasites has been scarce thus far. This research aimed to isolate filamentous fungi from avian fecal samples and evaluate their predatory activity against coccidia. A pool of 58 fecal samples from chickens, laying hens, and peacocks, previously collected between July 2020-April 2021, were used for isolation of filamentous fungi and assessment of their in vitro predatory activity against coccidian oocysts, using Water-Agar medium and coprocultures. The Willis-flotation technique was also performed to obtain concentrated suspensions of oocysts. A total of seven *Mucor* isolates was obtained, being the only fungal taxa identified, and all presented lytic activity against coccidia. Isolates FR3, QP2 and SJ1 had significant coccidiostatic efficacies (inhibition of sporulation) higher than 70%, while isolates FR1, QP2 and QP1 had coccidicidal efficacies (destruction of the oocysts) of 22%, 14% and 8%, respectively, after 14 days of incubation, being a gradual and time-dependent process. To our knowledge, this is the first report regarding the isolation of native predatory fungi from avian feces and demonstration of their lytic activity against coccidia.

## Introduction

Avian Coccidiosis is one of the most important parasitic diseases affecting domestic and exotic birds worldwide, being responsible for severe health and economic concerns in poultry farms, ornithological parks, and private bird collections^1–5^.

The control of this parasitic disease is mainly achieved through chemotherapy (e.g., anticoccidials) and vaccines. However, due to increasing concerns regarding antiparasitic drug resistance, extensive research has been conducted aiming at developing new alternative or complementary strategies to control Coccidiosis in bird collections, including feed improvement, house cleaning and disinfection, as well as natural solutions like herbal extracts, essential oils, probiotics and prebiotics, and algae^5–9^. More recently, Portuguese, Spanish, Brazilian and Danish researchers have been proposing the use of predatory fungi (also known as “nematophagous fungi” or “helmintophagous fungi”) with larvicidal and ovicidal characteristics as a complement to antiparasitic drugs for the control of gastrointestinal parasitic infections in domestic and exotic birds^10^.

The biocontrol of animal gastrointestinal parasitic infections using predatory fungi has already proved to be an accurate and sustainable complement to antiparasitic drugs, achieving efficacies of up to 97% in reducing the parasite egg shedding (number of eggs per gram of feces, EPG) in horses and ruminants^11–23^. However, only a few in vitro and in vivo studies have assessed the performance of predatory fungi against parasites affecting other animal hosts, namely birds, dogs, raccoons and wapitis^10,19,24–28^.

Predatory fungi are also known for their ubiquity, having been mostly isolated from agricultural soil, decaying organic matter, and animal feces^29^. Studies performed in America, Europe, Asia, Oceania and Antarctica have reported the isolation of filamentous fungi with ability to predate intestinal parasitic forms, from feces belonging to a wide diversity of animal species, including: sheep, goats and bovines^30–34^; water buffalo^35^; donkeys^34^; coati, raccoon, Eurasian lynx, Brown bear, mouflon, gazelle, bison, dromedary, guanaco and wallaby^33^; and horses^31,33^. The most commonly isolated taxa of predatory fungi with larvicidal properties are *Duddingtonia flagrans* (Dudd.) R.C. Cooke (1969), *Arthrobotrys* spp., and *Monacrosporium* spp., while *Pochonia chlamydosporia* (Goddard) Zare & W. Gams (2001), *Mucor circinelloides* Tiegh (1875), *Purpureocillium lilacinum* (Thom) Luangsa-ard, Houbraken, Hywel-Jones & Samson (2011), *Verticillium* spp., and *Trichoderma* spp. have shown to present ovicidal properties^17,29,36^. Nevertheless, studies on the isolation of these fungi from avian fecal samples have not yet been reported in the scientific literature.

The current research aimed to isolate native filamentous fungi from fecal samples of domestic and exotic birds and assess its in vitro predatory activity on coccidian oocysts.

## Results

### Fungi isolation and identification

From the pool of 58 feces belonging to chickens, laying eggs, and peacocks, it was possible to obtain seven isolates of filamentous fungi: three from chickens (FR1, FR2 and FR3) and four from peacocks (SJ1 and SJ2 – exotic bird collection SJ; QP1 and QP2 – exotic bird collection QP).

**Figure 1:**
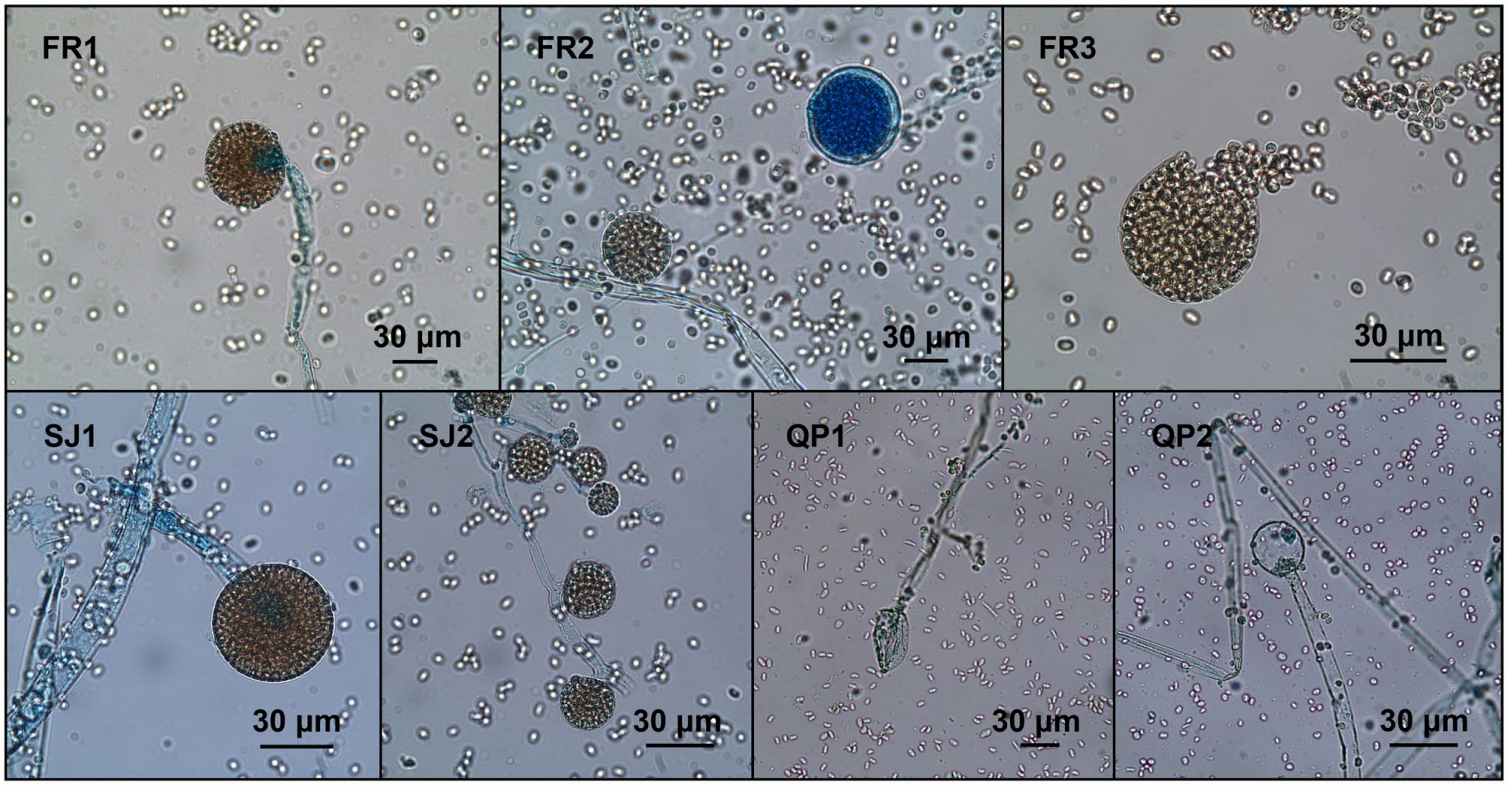
Conidia, sporangia, and hyphae of *Mucor* isolates (FR1—*M. circinelloides*; FR2—*M. circinelloides*; FR3—*M. circinelloides*; SJ1—*M. circinelloides*; SJ2—*M. circinelloides*; QP1—*M. lusitanicus*; QP2—*M. circinelloides*; originals).

Macroscopic and microscopic fungal characterization revealed similar results for most isolates: ovoidal and hyaline conidia, without septa; yellowish and non-branched sporangia, supported by a columella; hyphae without septa; grey-white and fluffy colonies. However, the isolate QP1 had conidia with an oblong shape, and hyphae thinner than the other isolates (Fig. 1; Table 1). Thus, morphological assessment allowed to presumptively identify all isolates as *Mucor* sp. No other fungal taxa were identified.

**Table 1:** Mean (± standard error) measures for conidia and sporangia length and width, and hyphae length.

Isolates	Conidia		Sporangia		Hyphae length
	Length (µm)	Width (µm)	Length (µm)	Width (µm)	(µm)
FR1	4.9 ± 0.3	3.7 ± 0.3	48 ± 3.4	47 ± 3.4	11 ± 1.3
FR2	5.5 ± 0.3	4 ± 0.3	45 ± 3.8	41 ± 3.8	11 ± 0.7
FR3	6.6 ± 0.3	4.4 ± 0.2	47 ± 3.7	46 ± 3.2	13 ± 1.1
SJ1	4.5 ± 0.3	3.4 ± 0.4	45 ± 4.2	42 ± 3.1	10 ± 1.1
SJ2	4.3 ± 0.2	3.1 ± 0.2	27 ± 2.0	26 ± 1.8	12 ± 1.5
QP1	4.6 ± 0.2	2.9 ± 0.2	30 ± 3.2	27.5 ± 3.1	6.8 ± 1.4
QP2	5.4 ± 0.5	3.8 ± 0.3	26.5 ± 2.3	24.5 ± 2.3	6.3 ± 1.5

The upload of ITS1-5.8S-ITS2 sequences to Blastn Suite allowed to establish a Top 3 of similarity for each fungal isolate: FR1 had over 99.5% similarity with two *Mucor circinelloides* strains (GenBank accession numbers OW988287 and OW985400); FR2 had over 99% similarity with three *M. circinelloides* strains (FN598920, HQ914900, and KJ584557); FR3 had 99.8% similarity with *two M. circinelloides* strains (MK396486 and KT336541); SJ1 had 100% similarity with three *M. circinelloides* strains (KX620480, OW987678, and OW987665); SJ2 had over 99.5% similarity with three *M. circinelloides* strains (MT991775, NR_126116, and FJ713065); QP1 had 99.8% similarity with *Mucor* sp. (MK164174), *Mucor lusitanicus* (OP163597) and *Mucor racemosus* (MN726736); and QP2 had 100% similarity with two *M. circinelloides* strains (MT603934 and OW988287) (Table 2).

**Table 2:** Similarity and query coverage percentages of each isolate sequence, in comparison with reported strains (Blastn).

Isolates Reported strains	Similarity (%)	Query coverage (%)	GenBank accession nr
Isolate FR1			
* Mucor circinelloides* IBT2M2	99.65	93	OW988287
* M. circinelloides*	99.49	95	OW985400
* Mucor* sp. 033b	98.48	93	MW789352
Isolate FR2			
* M. circinelloides* IBT2H2	99.31	93	FN598920
* M. circinelloides* OUCMBI101096	99.15	96	HQ914900
* M. circinelloides* Sz8H	99.15	96	KJ584557
Isolate FR3			
* M. circinelloides* MDM14	99.83	96	MK396486
* M. circinelloides* M37	99.83	96	KT336541
* Mucor* sp. BAB-4784	99.83	96	KR154996
Isolate SJ1			
* M. circinelloides* AW1085	100	95	KX620480
* M. circinelloides*	100	95	OW987678
* M. circinelloides*	100	95	OW987665
Isolate SJ2			
* M. circinelloides* JEHAN37	99.83	97	MT991775
* M. circinelloides* CBS195.68	99.83	97	NR_126116
* M. circinelloides* E2A	99.67	97	FJ713065
Isolate QP1			
* Mucor lusitanicus* WZ-900	99.83	94	OP163597
* Mucor racemosus* GZ20190123	99.83	94	MN726736
* Mucor* sp. REB-039A	99.83	94	MK164174
Isolate QP2			
* M. circinelloides* CMRC545	100	96	MT603934
* M. circinelloides*	100	96	OW988287
* Mucor* sp. F8-2018	100	96	MW789352

**Figure 2:**
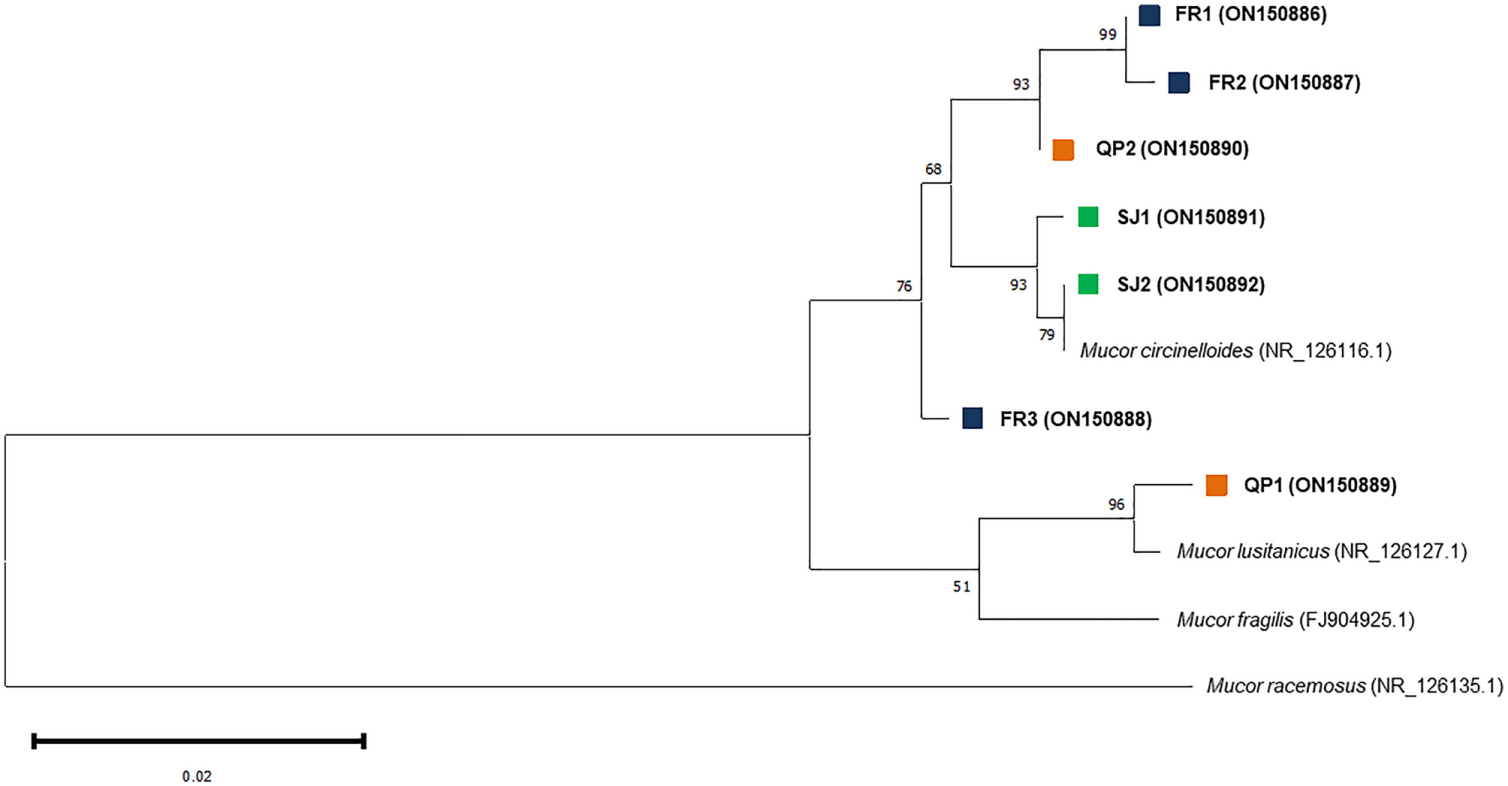
Maximum likelihood phylogenetic tree based on ITS1-5.8S-ITS2 region, including isolates’ and GenBank reference strains’ sequences. The likelihood’s bootstrap is shown in each branch, and only values above 50 were considered. All seven *Mucor* isolates are displayed in bold, followed by their respective GenBank accession numbers. Coloured squares represent the bird collections from which fungi were obtained: blue—chickens; green and orange—peacocks from exotic collections SJ and QP, respectively.

Phylogenetic analysis of the ITS1-5.8S-ITS2 sequences of all isolates, using the strains *Mucor circinelloides* CBS 195.68, *Mucor lusitanicus* CBS 108.17, *Mucor racemosus* f. *racemosus* CBS 260.68 and *Mucor fragilis*, obtained also from BLAST analysis, allowed to identify isolates FR1, FR2, FR3, SJ1, SJ2 and QP2 as *Mucor circinelloides* rDNA sequences, whereas the isolate QP1 was identified as *Mucor lusitanicus* (Fig. 2).

### In vitro biocontrol trials

**Figure 3:**
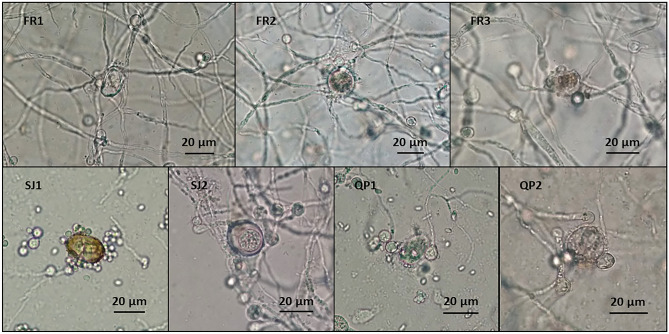
Predation developed by *Mucor* isolates against coccidian oocysts, in WA medium (FR1—*M. circinelloides*; FR2—*M. circinelloides*; FR3—*M. circinelloides*; SJ1—*M. circinelloides*; SJ2—*M. circinelloides*; QP1—*M. lusitanicus*; QP2—*M. circinelloides*; originals).

**Figure 4:**
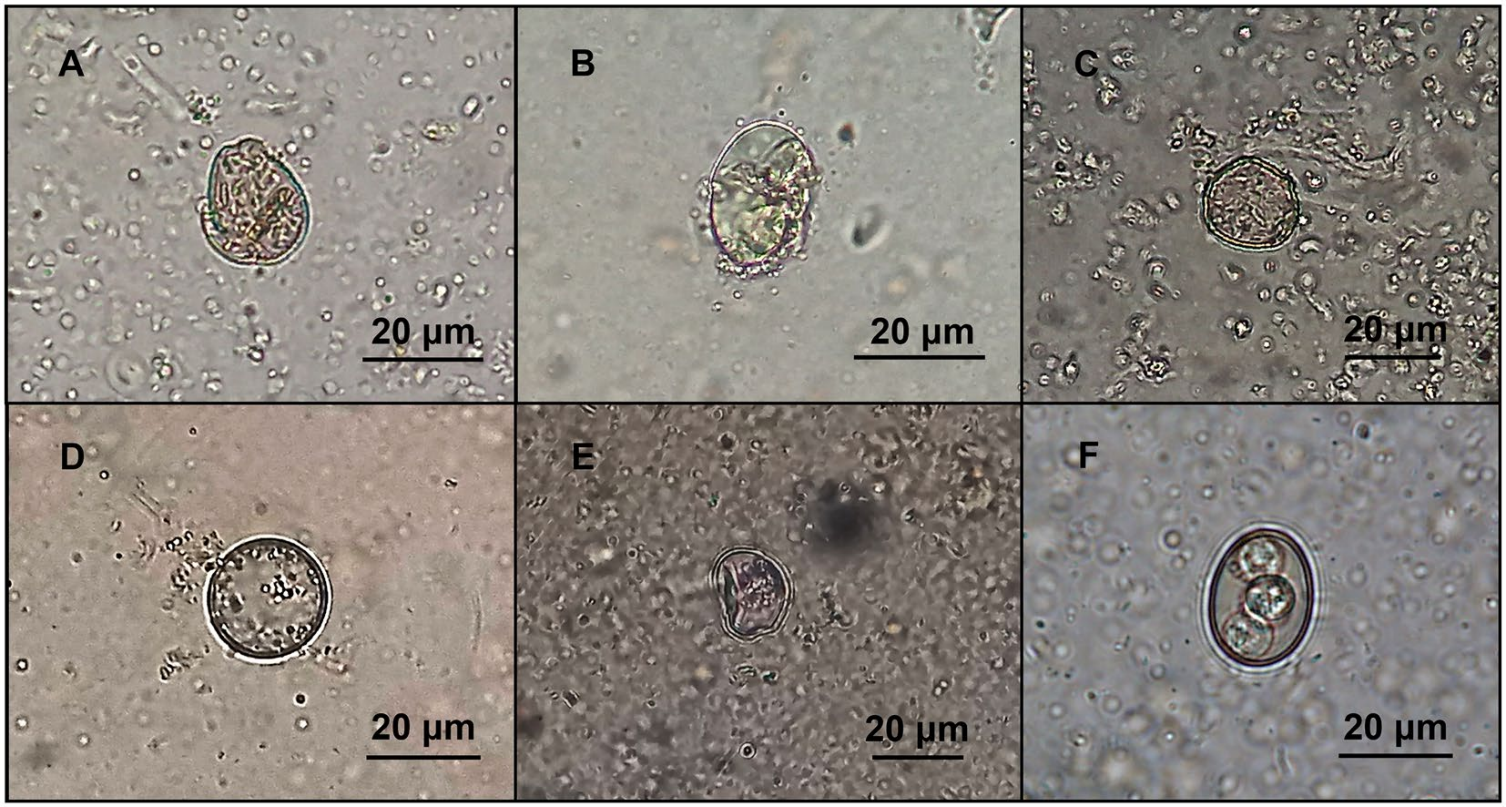
*Eimeria* sp. oocysts showing different morphological changes, following coprocultures with fungi (**A**, **C**, **E**—oocyst’s capsule deformation; **B**, **D**—oocyst disruption and loss of cytoplasmic content; **F**—sporulated oocyst; originals).

In vitro trials revealed that all isolates developed predacious activity against *Eimeria* oocysts, both in WA medium (Fig. 3) and in coprocultures (Fig. 4). It was possible to observe that the presence of oocysts triggered the development of fungi hyphae and their adhesion to the oocysts’ capsules (activity type 1). Also, during 30 days of fungal exposure, oocysts started to change their morphology, showing their inner structures poorly marked, and developing vacuoles (type 2), until spores started proliferating within the oocyst cell and finally leading to its disruption (type 3).

Fungal isolates showed different lytic performances when exposed to the fecal microenvironment (plastic cups trial), in terms of reducing coccidia sporulation and damaging the oocysts structure: isolates FR3, QP2, SJ1, SJ2 and FR2 had significant reduction efficacies of 85% (*p* < 0.001), 85% (*p* < 0.001), 73% (*p*  = 0.001), 69% (*p* = 0.001) and 65% (*p* = 0.003), respectively, on limiting the sporulation of oocysts, while isolates FR1, QP2 and QP1 had significant reduction efficacies of 22% (*p* < 0.001), 14% (*p* < 0.001) and 8% (*p* = 0.01), respectively, on destroying the oocysts, but only after 14 days of exposure. Also, the ovicidal efficacy was time-dependent, since the oocyst viability differed significantly between the first and second weeks of the trial, after exposure to the isolates FR1 (*p* < 0.001) and QP1 (*p*  = 0.001). The oocysts viability remained stable in the control cup during the assay, with percentages of viability equal to 97% and 94%, after 1 and 2 weeks of incubation, respectively (Table 3), with no statistical differences being recorded between both weeks (*p* = 0.302).

**Table 3:** Quantification of the predacious activity developed by each fungal isolate against coccidia. Sporulated and viable oocyst percentage reads (R1 and R2) and its mean and standard errors (SE) are provided, as well as the average reduction in oocyst sporulation (FOSR) and viability (FOVR) in comparison with control, after 7 and 14 days of incubation; significant results (*p* < 0.05) are highlighted in bold and marked with an asterisk.

Isolates	FOSR					FOVR									
	7 days					7 days					14 days				
	R1 (%)	R2 (%)	Mean ± SE (%)	Reduction (%)	*p*	R1 (%)	R2 (%)	Mean ± SE (%)	Reduction (%)	*p*	R1 (%)	R2 (%)	Mean ± SE(%)	Reduction (%)	*p*
FR1	8	10	9 ± 1	31	0.20	92	95	93.5 ± 1.5	3	0.17	67	81	74 ± 7	22*	< 0.001
FR2	5	4	4.5 ± 0.5	65*	0.003	94	96	95 ± 1	2	0.46	92	93	92.5 ± 0.5	2	0.47
FR3	1	3	2 ± 1	85*	< 0.001	91	95	93 ± 2	4	0.12	97	90	93.5 ± 3.5	1	0.73
SJ1	4	3	3.5 ± 0.5	73*	0.001	98	98	98	0	-	90	92	91 ± 1	3	0.21
SJ2	3	5	4 ± 1	69*	0.001	94	94	94	3	0.24	93	90	91.5 ± 1.5	3	0.27
QP1	7	11	9 ± 2	31	0.20	98	95	96.5 ± 1.5	0	-	87	87	87	8*	0.01
QP2	2	2	2	85*	< 0.001	94	92	93 ± 1	4	0.12	82	81	81.5 ± 0.5	14*	< 0.001
Control	15	11	13 ± 2	-	-	97	96	96.5 ± 0.5	-	-	93	96	94.5 ± 1.5	-	-

## Discussion

Predatory fungi are a group of saprophytic filamentous fungi known for their ability to predate and destroy larvae, eggs and oocysts from parasites affecting animals and plants. Besides these functional characteristics, they are also known for other attributes, namely the possibility to be isolated from a wide diversity of environmental samples, including agricultural soil, decaying organic matter and animal feces. Their isolation from fecal matter, which frequently also harbours environmental forms of intestinal parasites, proves that fungi and parasites naturally establish relationships in the fecal and soil microenvironment, with the formers being a nutritional source for predatory fungi^17,29,36^. Also, the isolation of this type of fungi from feces belonging to healthy animals demonstrates the equilibrium in which these microorganisms are within the intestinal environment, and thus their innocuity to immunocompetent animals^17,19,20,28,37–39^.

To our knowledge, this study allowed to isolate for the first time filamentous fungi with predatory capacities from bird fecal samples, suggesting that birds are also “natural shedders” of this kind of fungi, as previously reported by several authors for mammal species, namely ruminants, horses and carnivores kept in farms and zoological parks^30–35^.

For this research, the use of avian fecal samples positive for intestinal parasites, together with their initial inoculation on a poor medium like Water-Agar, allowed to restrict the groups of fungi able to develop in this medium and stimulate the growth of only potential predatory fungi. Also, besides WA medium, the isolation steps also featured Wheat-flour Agar for rapid hyphae growth, purification, and storage^12^. The use of these two media, with no antibiotic supplementation, allowed to accurately isolate and store predacious fungi, in a quicker and economical approach in comparison with other more nutritive mediums like Sabouraud Agar, Corn Meal Agar, Potato Dextrose Agar or Malt Extract Agar, which are often supplemented with Chloramphenicol for these procedures.

Filamentous fungi isolates were obtained in all selected locations and from the two bird model species used, despite no isolates being obtained from laying hens’ samples. Morphological analysis allowed to conclude that all isolates belong to the genus *Mucor*, with qualitative and quantitative results tracked for conidia, sporangia and hyphae being in accordance with published literature regarding this genus^40,41^. Also, molecular assessment based on rDNA ITS1-5.8S-ITS2 sequences led to the identification of two fungal species, *Mucor circinelloides* (FR1, FR2, FR3, SJ1, SJ2 and QP2) and *Mucor lusitanicus* (QP1), and thus proving that these target sequences are indeed suitable to be used in the molecular identification of predatory fungi, as demonstrated in other studies^34,42–45^.

All fungal isolates developed lytic activity against coccidian oocysts in WA medium and within the fecal microenvironment, allowing to identify all stages of predatory activity. Regarding the first assay, predating efficacies differed between strains, with FR1 and QP2 having been the most accurate on destroying *Eimeria* spp. oocysts (efficacies of 22% and 14%, respectively), while strains FR3, QP2 and SJ1 presented significant coccidiostatic efficacies, higher than 70%. These results are in accordance with previous studies performed by Portuguese and Spanish researchers^27,33^, which demonstrated in vitro predatory activity developed by *M. circinelloides* against eggs and oocysts from intestinal parasites affecting different animal hosts. Moreover, it constitutes the first original research article reporting the coccidicidal and coccidiostatic activity of *Mucor* spp. against avian coccidia. Also, the current research reveals for the first time the predatory skills developed by *M. lusitanicus* against parasitic forms, which had a significant impact on the oocysts viability after 14 days of incubation (8%), despite presenting an efficacy lower than the ones obtained for the other strains. The ability of *Mucor* spp. to predate avian intestinal parasitic forms is one of the research lines of Spanish and Portuguese authors belonging to the COPAR research group (Faculty of Veterinary – University of Santiago de Compostela) and the LPPD (Faculty of Veterinary Medicine – University of Lisbon), respectively.

The detection of significant results for fungal coccidicidal activity only after 14 days of incubation, and the significant differences between data from 7 and 14 days, for isolates FR1 and QP1, confirms that the predatory activity developed by this kind of fungi is a gradual and time-dependent process. It is the presence of the parasite that triggers the development of fungi hyphae towards it and their adhesion to its capsule^17,27,29,33,36^. Fungal hyphae migration towards parasite eggs and oocysts may be considered as one of the most critical stages of the predatory process. Hyphae must grow and reach the parasite, which is a process that can be delayed by native biotic and abiotic factors within the fecal microenvironment, and thus affecting the performance and speed of fungal action. The avian fecal microbiota, which is composed by a wide diversity of native microorganisms, namely bacteria of the Phyla Firmicutes and Proteobacteria^46^, fungi of the Phyla Ascomycota and Basidiomycota^47^, and other microorganisms, may have negatively influenced the performance of each fungal strain, due to resource competition and degradation of the fungal strains. It has been suggested that predatory fungi survival within the soil and fecal microenvironment is affected by biotic factors such as the presence of microorganisms with fungistatic characteristics^48^. Furthermore, a study performed in Denmark^49^ demonstrated that the in-soil predatory performance of *Pochonia chlamydosporia* and *Metarhizium brunneum*, two ovicidal fungi species, against avian ascarid eggs (*Ascaridia galli* and *Heterakis gallinarum*), is affected by the soil’s microbiota. All these factors need to be considered when planning a biocontrol assay, namely the optimal dosage of spores to counter these limiting factors.

Finally, since all *Mucor* strains were isolated from avian fresh fecal samples, and showed interesting lytic activity on coccidian oocysts, it can be suggested that these strains resisted to the gastrointestinal passage in chickens and peacocks, and maintained both their germination and predatory capacities, as previously demonstrated in birds for the ovicidal fungus *P. chlamydosporia*^50^ and larvicidal fungi *D. flagrans* and *Monacrosporium thaumasium*^51^. The fact that all *Mucor* isolates were obtained from feces belonging to healthy birds, allows also to suggest their innocuity to immunocompetent birds, as demonstrated by other researchers for horses^37^, sheep^20^, dogs^28^ and wapitis^19^.

To our knowledge, this study was the first performed worldwide aiming to isolate and identify native predatory fungi from bird feces and test their in vitro efficacy against avian *Eimeria* spp. oocysts. Results suggest that *Mucor circinelloides* strains FR1 and QP2 are the most promising to be used in future in vitro and in vivo biocontrol trials.

## Methods

### Fecal samplings and coprological analysis

A total of 89 fecal samples from free-range chickens and laying hens (*Gallus gallus domesticus*; n = 46) and peacocks (*Pavo cristatus*; n = 43), were previously assessed using several coprological techniques, such as McMaster, Mini-FLOTAC and Coprocultures, in the scope of a recent study performed at the Laboratory of Parasitology and Parasitic Diseases (LPPD) of Faculty of Veterinary Medicine, University of Lisbon ^3^, which reported that 58 samples (65%) were positive for at least one gastrointestinal parasite taxon, namely coccidia of the genus *Eimeria*, and nematodes like *Capillaria* sp., *Trichostrongylus tenuis* and *Strongyloides pavonis*. Samples belonged to healthy animals, which did not show any clinical signs of gastrointestinal disorders, namely diarrhoea and/or feces with blood.

These samples were collected between July 2020 and April 2021, in a poultry farm (PF) and two exotic bird collections located in Lisbon (SJ) and Santarém (QP) districts (Portugal). The poultry farm is located in North-western Lisbon (39°13′54.373″ N 9°17′2.235″ W) and harbours two separate populations of 200 free-range chickens and 200 laying hens, while the exotic bird collections SJ and QP have 20 and 3 peacocks, being located in central Lisbon (38°42′50.241″ N 9°8′2.182″ W) and Abrantes (39°26′52.595″ N 8°10′24.949″ W), respectively.

Fecal samples were immediately collected after excretion, packed in plastic bags, and transported to LPPD, being stored in a refrigerator (4 °C) for a maximum length of 1 week, until further processing.

### Isolation and morphological identification of filamentous fungi

A total of 58 avian fecal samples positive for gastrointestinal parasites were used for isolation and identification of filamentous fungi, at both the LPPD and the Laboratory of Mycology of the Faculty of Veterinary Medicine – University of Lisbon (Portugal). The idea of using only these samples was based on the premise that this kind of fungi have as main ability the predation and destruction of parasite eggs, oocysts and larvae, and thus this procedure would stimulate the growth of potential predaceous fungi and restrict the development of other fungal groups.

For this purpose, approximately 1 g of each fecal sample was placed on the surface of Water-Agar medium (WA, 2%), and then incubated at 26 °C for 3 weeks. Once filamentous fungi growth was recorded, individual colonies were subjected to 3–4 passages, using Wheat-Flour Agar (WFA, 2%) and incubation cycles of 26 °C for 1 week, until achieving pure cultures. Two replicates were used for each fecal sample^33^.

All isolates were subjected to morphological identification at the genus level, based on Hernández et al.^33^, Arroyo-Balán et al.^34^, Ocampo-Gutiérrez et al.^42^ and Cooke and Godfrey^52^. Measurements (length and width) and morphology description were carried out for a total of 10 sporangia and hyphae (200X and 400X total magnification), and conidia (1000X total magnification, in immersion oil), using a lactophenol cotton blue stain and a light microscope. Also, macroscopical characterization of the colonies was performed for each isolate, regarding its texture and colour.

Suspensions of spores were established for each isolate using distilled-water, and their final concentration was calculated using the Neubauer chamber. All fungal suspensions were standardized to 10^6^ spores/mL.

Fungal isolates were preserved in Petri dishes and glass flasks with WFA at room temperature, and 850 μL of each fungal aqueous suspension were stored at − 20 °C in cryotubes with 15% (v/v) sterile glycerol^40^.

### Molecular characterization of fungal isolates

#### DNA extraction

DNA extraction from all fungal isolates was performed using the E.Z.N.A.® Fungal DNA Mini kit (Omega Bio-Tek, Norcross, GA). A calibrated 1 μL swab was used to collect fresh mycelia from each fungal isolate. This procedure was repeated 5 times, and the total mycelia volume was placed in the respective 2 mL microcentrifuge tubes, to which 600 μL of lysis buffer FG1 were also added. The mixture was vortexed to disperse all clumps and incubated at 65 °C for 10 min. Then, 140 μL of FG2 buffer (glacial acetic acid) were added, and the suspension was vortexed. Tubes were incubated on ice for 5 min, and then centrifuged at 10,000 × g for 10 min. Supernatants were transferred to new microcentrifuge tubes, to which were also added 0.7 volumes of isopropanol. After vortex, suspensions were centrifuged at 10,000 × g for 2 min. The supernatants were discharged, and 300 μL of sterile distilled water were added to each DNA pellet, and then vortexed. A total of 4 μL of RNase A was added to each tube, followed by 150 μL of FG3 buffer (guanidine hydrochloride) and 300 μL of 100% ethanol, always using the vortex to mix the suspensions. Further steps were performed using HiBind® DNA Mini Columns to eliminate polysaccharides, phenolic compounds, and enzyme inhibitors from fungal lysates, due to its reversible nucleic acid-binding. Pure DNA was eluted in 200 μL of sterile distilled water, and its purity and concentration were checked using NanoDrop™ (Thermo Fisher Scientific Inc., Waltham, USA). Tubes were finally stored at -20 °C.

#### Amplification of ribosomal DNA

Amplification of rDNA was performed for each isolate targeting the ITS1-5.8S-ITS2 region, using 10–66 ng of genomic DNA and primers ITS1 (TCC GTA GGT GAA CCT GCG G) and ITS4 (TCC TCC GCT TAT TGA TAT GC). These procedures followed the guidelines described by Arroyo-Balán et al.^34^ and Lau et al.^53^, and the PCR reaction was performed in a 25 μL volume, composed by: 0.4 μL ITS1 (0.8 μM), 0.4 μL ITS4 (0.8 μM), 10 μL DNA template, 10 μL NZYTaq II Green Master Mix (NZYTech, Lisbon, Portugal) and 4.2 μL molecular biology water. A negative control was also used, by replacing DNA for water.

Thermocycling conditions were the following: an initial denaturation step at 95 °C for 10 min, followed by 60 cycles composed by a denaturation step at 94 °C for 15 s, an annealing step at 55 °C for 30 s, and an extension step at 72 °C for 30 s. Finally, an extension step was performed at 72 °C for 5 min. The amplicons were analysed by agarose-gel (1.5%) electrophoresis, stained with 2.5 μL of GreenSafe Premium (NZYTech), and including the NZYDNA Ladder VI (50–1500 bp; NZYTech). The gel was run at 85 V for 40 min and visualized using the equipment ChemiDoc and the Image Lab™ Software (Bio-Rad Laboratories, Inc., California, USA).

Each PCR product was purified using magnetic beads (MCLAB, California, USA) for DNA precipitation, followed by a pellet wash with 85% ethanol and subsequent elution in MiliQ water. The obtained supernatants were used for further sequencing. Purified PCR products were sequenced using the ITS1 primer and BigDye™ Terminator version 3.1 Cycle Sequencing Kit, and also with the equipment DNA Analyzer 3730 XL (Thermo Fisher Scientific Inc.).

Sequences were assessed using Chromas Software, version 2.6.6 (Technelysium Pty, Ltd., South Brisbane, Australia), and their quality was checked based on well-defined peaks of the nucleotides in the chromatograms. Sequences were then blasted using the Blastn Suite (BLAST®) of the National Centre for Biotechnology Information (NCBI), to perform a preliminary analysis of sequences significant alignments and select fungal taxa for comparative purposes in the phylogenetic analysis. For each fungal isolate, a Top 3 of sequence similarity was established based on the results from Blastn Suite.

#### Phylogenetic analysis

Sequences were manually edited using MEGA software, version 11.0.11^54^, to check for the quality of sequences and undetected nucleotides, remove primers, as well as to perform their alignment using the MUSCLE algorithm. Based on the BLAST® search, ITS1-5.8S-ITS2 region sequences from isolates *Mucor circinelloides* CBS 195.68 (accession number: NR_126116.1), *Mucor lusitanicus* CBS 108.17 (accession number: NR_126127.1), *Mucor racemosus* f. *racemosus* CBS 260.68 (accession number: NR_126135.1) and *Mucor fragilis* (accession number: FJ904925.1) were also included. The IQ-TREE web server^55^ was used to generate a maximum likelihood phylogenetic tree from 1000 replications. The ModelFinder option of IQ-TREE was set for auto-determination of the best model^56^. The ultrafast bootstrapping tool (1000 bootstrapped alignments) was chosen to obtain node support statistics, with its branches being only supported by bootstrap values above 50^57^. The phylogenetic tree was visualized and edited using MEGA software, and *M. racemosus* was chosen to root the tree, since in the initial maximum likelihood phylogenetic tree, this reference strain was clearly more phylogenetically distant from the other fungal isolates and reference strains, and both rooting the tree on this strain and on Midpoint resulted in identical phylogenetic trees.

The ITS1-5.8S-ITS2 nucleotide sequences of all seven fungal isolates were deposited in the GenBank database under the following accession numbers: ON150886 (*M. circinelloides*, FR1), ON150887 (*M. circinelloides*, FR2), ON150888 (*M. circinelloides*, FR3), ON150889 (*M. lusitanicus*, QP1), ON150890 (*M. circinelloides*, QP2), ON150891 (*M. circinelloides*, SJ1) and ON150892 (*M. circinelloides*, SJ2).

### In vitro biological control trials against avian coccidia

Two types of biocontrol trials were conducted aiming to test the predatory activity of all fungal isolates against avian coccidia: a qualitative assay in Petri dishes containing WA medium, and a quantitative–qualitative coproculture assay^33^.

To obtain concentrated suspensions of oocysts, fecal samples from chickens, laying hens and peacocks, positive for *Eimeria* spp., were processed using the Willis-flotation technique. Briefly, two grams of feces were mixed with 28 mL of saturated sucrose solution (specific gravity 1.2); the fecal suspension was filtrated and poured to 10 mL test tubes, until the formation of a convex meniscus, on which a coverslip was placed; test tubes were left on the lab bench for 10 min, and the coverslip was then washed with distilled water to new test tubes, which were centrifugated at 2000 rpm for 10 min; the supernatant was partially removed, leaving just 1 mL in each tube; the sediment and supernatant were mixed using a Pasteur pipet, and 100 μL of the oocysts suspension were visualized using a light microscope, at 400X total magnification. Two reads were performed and the total oocyst count was multiplied by 10 to calculate the coccidia concentration (i.e., oocysts/mL).

In the first assay, a total volume of 500 μL of each fungal isolate (10^6^ spores/mL) were inoculated on the surface of WA medium, to which 1 mL of oocyst suspension was also added, with a mean concentration of 140 oocysts/mL. Two replicates were used for each isolate, and a positive control was also used to assess the survival of the oocysts without fungal inoculate and test contamination by other fungal species. Plates were sealed with Parafilm and incubated at room temperature for 30 days. Then, plates were observed for identification of predatory activity, which was characterized as follows: hyphae attachment to the oocysts capsule but without morphological damage (activity type 1); the oocysts capsule and inner structures exhibiting morphological changes, but without fungal penetration (type 2); hyphae penetrate into the oocyst cytoplasm, grow inside, and destroy it (type 3)^27,33,58^.

The second assay aimed to evaluate the fungal isolates efficacy on degrading the oocysts, following exposure to the fecal microenvironment. Four grams of peacock fecal samples from the exotic collection SJ, positive for *Eimeria* spp. (n = 20), were gently mixed and placed in eight plastic cups. A total of 4 mL of fungal suspensions (10^6^ spores/mL) were added to the respective test cups (one per fungal isolate, n = 7), while 4 mL of distilled water were poured onto the control cup (n = 1). Then, cups were covered with perforated aluminium foil and left incubating for two weeks, at 26 °C. After one and two weeks of incubation, two flotations were performed in each test and control cups, using 2 g of feces randomly picked from distinct parts of the sample and mixing it with 28 mL of saturated sucrose solution (specific gravity 1.2), aiming to calculate the proportion of sporulated/unsporulated and viable/unviable oocysts (after one week) and the proportion of viable/unviable oocysts (in each week). Two reads were performed in each cup and timeframe, by counting a total of 100 oocysts per read.

For each fungal isolate and timeframe (7 and 14 days), the fecal oocyst viability reduction (FOVR) (1) and fecal oocyst sporulation reduction (FOSR) (2) were calculated as follows^19,20,59^:

1 $${\text{FOVR }}\left( \% \right){\kern 1pt} = {\kern 1pt} \left[ {1 - \left( {{\text{VIABILITY}}\;{\text{test / VIABILITY}}\;{\text{control}}} \right)} \right]{\kern 1pt} \times {\kern 1pt} 100$$

2 $${\text{FOSR }}\left( \% \right){\kern 1pt} = {\kern 1pt} \left[ {1 - \left( {{\text{SPORULATION}}\;{\text{test / SPORULATION}}\;{\text{control}}} \right)} \right]{\kern 1pt} \times {\kern 1pt} 100$$

The characterization of the oocysts appearance was adapted from the procedures established for ascarid eggs by Cazapal-Monteiro et al.^27^, with oocysts being considered as unviable if at least one of the following characteristics was observed: inner structures poorly marked, oocysts abnormal shape, cytoplasm containing vacuoles, and/or capsule disruption.

Also, since most *Eimeria* species affecting Galliformes sporulate in less than 2 days, at temperatures ranging between 20 and 30 °C^5,60^, the FOSR assessment was performed according to the following criteria: at the end of the first week of incubation, the identification of non-sporulated oocysts in the test cups was attributed to a coccidiostatic activity developed by the exposure to the respective fungal isolates.

### Statistical analysis

The software Microsoft® Excel® for Microsoft 365 MSO (Microsoft Corporation, Redmond, WA, USA), was used for data storage, and table and chart editing.

The software IBM® SPSS® Statistics version 27 for Windows (IBM Corporation, Armonk, NY, EUA) was used for the initial descriptive statistics (mean and standard errors). Also, this software was used to build 2 × 2 tables using data from the in vitro trial (viability and sporulation), aiming at performing a Chi-Square test, to compare the results obtained between the oocysts exposed to each fungal isolate (test cups) and to water (control cup). Moreover, this test was used to assess the time-dependency of the ovicidal activity developed by each fungal isolate on oocysts. A significance level of *p* < 0.05 was used for all tests.

## Data Availability

The datasets generated and/or analysed during the current study belong to the Centre for Interdisciplinary Research in Animal Health (CIISA), Faculty of Veterinary Medicine, University of Lisbon. rDNA sequences obtained from all seven *Mucor* isolates were uploaded to the GenBank database, on 5 April 2022, with the accession numbers being provided in the Methods section. All data can be made available by requesting it from the Corresponding Author.
